# The variation of functional connectivity and activity before and after thalamotomy surgery (review)

**DOI:** 10.3389/fnhum.2023.1108888

**Published:** 2023-04-28

**Authors:** Mohammad-Hossein H. K. Nili, Shahrzad M. Esfahan, Yamin Bagheri, Abdol-Hossein Vahabie, Mehdi Sanayei, Abolhassan Ertiaei, Mohammad Shirani, Mohammad-Reza A. Dehaqani, Ehsan Rezayat

**Affiliations:** ^1^School of Electrical and Computer Engineering, College of Engineering, University of Tehran, Tehran, Iran; ^2^Department of Psychology, Faculty of Psychology and Education, University of Tehran, Tehran, Iran; ^3^School of Cognitive Sciences, Institute for Research in Fundamental Sciences (IPM), Tehran, Iran; ^4^Institute for Cognitive Science Studies (ICSS), Tehran, Iran; ^5^Department of Neurosurgery, Sina Hospital, Tehran University of Medical Science, Tehran, Iran

**Keywords:** ablation surgery, thalamotomy, functional connectivity, activity, fMRI, EEG

## Abstract

Ablation surgeries are utilized to treat certain brain disorders. Recently, these surgeries have become more prevalent using techniques such as magnetic resonance guided focused ultrasound (MRgFUS) ablation and Gamma knife thalamotomy (GKT). However, as the thalamus plays a critical role in cognitive functions, the potential impact of these surgeries on functional connectivity and cognition is a matter of concern. Various approaches have been developed to locate the target for ablation and also investigate changes in functional connectivity before and after surgery. Functional magnetic resonance imaging (fMRI) and electroencephalogram (EEG) are widely used methods for assessing changes in functional connectivity and activity in clinical research. In this Review, we summarize the use of fMRI and EEG in thalamotomy surgeries. Our analysis shows that thalamotomy surgery can result in changes in functional connectivity in motor-related, visuomotor, and default-mode networks, as detected by fMRI. EEG data also indicate a reduction in over-activities observed in the preoperative state.

## Introduction

The thalamus is a critical node in the brain network and acts as a hub in information transmission (Tomasi and Volkow, 2011). This area is extensively connected to the cerebral cortex via thalamocortical radiations (Coenen et al., 2014). This massive connectivity means that the thalamus plays a vital role in many cognitive, sensory, and executive functions (Hwang et al., 2017). Manipulating the thalamus, such as through thalamotomy, can have a significant impact on behavior and the brain networks (Halpern et al., 2019; Krishna et al., 2020; Lin et al., 2022), making it an effective treatment for essential tremor (ET), Parkinson’s disease (PD) (Zaaroor et al., 2017), Holmes tremor (HT) (Kim et al., 2002), epilepsy (Sitnikov et al., 2016), neurogenic pain (Sarnthein et al., 2006), multiple sclerosis (MS) (Mathieu et al., 2007), and dyskinesia (Lee, 1997). Monitoring the effects of thalamotomy surgery on brain function is essential to evaluate the success of procedure, predict patient outcomes, and adjust treatment plans accordingly.

Thalamotomy is an effective method to decrease dominant disease symptoms, such as tremors, unwanted motor activity, poor quality of life, and seizures (Lipsman et al., 2013). The first thalamotomy surgery have done using the stereotactic approach was a pallido- thalamotomy, which Spiegel and Wycis carried out in 1946 on a patient with Huntington’s Disease (HD) to reduce emotional reactivity by targeting the medial nucleus of the thalamus (Ea and Ht, 1950; Sarnthein et al., 2006). Today, using new thalamotomy methods such as, Magnetic resonance guided focused ultrasound (MRgFUS) (Stanziano et al., 2022), and gamma knife thalamotomy (GKT) (Tuleasca et al., 2021) these surgeries have become prevalent. However, the system-level description of thalamotomy effectiveness remains rudimentary. The improvement resulting from thalamotomy surgery is thought to arise from changing the activity and the functional connectivity in the associated regions (Jang et al., 2016). To evaluate the functional connectivity and activity in the brain network before and after thalamotomy, it is necessary to use methods such as functional magnetic resonance imaging (fMRI) and electroencephalography (EEG) (Tuleasca et al., 2018d), beside of other methods such as positron emission topography (PET), magnetoencephalography (MEG) and functional near-infrared spectroscopy (fNIRS). fMRI and EEG is currently the dominant methods due the superior spatial resolution of fMRI and the temporal resolution of EEG, we also evaluate several studies that used other methods during thalamotomy surgery.

Combining EEG and fMRI in preoperative and postoperative conditions will enable a comprehensive evaluation of the functional connectivity and activity during thalamotomy. Although simultaneous EEG-fMRI studies exist (Abreu et al., 2019; Boerwinkle et al., 2022), there is a lack of studies that compare pre and post-thalamotomy conditions. Therefore, the objective of this study is to summarize the application of EEG and fMRI in thalamotomy surgeries. Analysis of the EEG in pre-and postoperative conditions shows a decrease in EEG over-activities after surgery, while, fMRI studies demonstrate changes in functional connectivity, particularly in motor (Hesselmann et al., 2006; Jang et al., 2016; Park et al., 2017; Tuleasca et al., 2021), visuomotor (Tuleasca et al., 2018a,c, 2020; Xiong et al., 2022a), and default-mode networks (Wen et al., 2016; Tuleasca et al., 2018b,e).

## Methods

**FIGURE 1:**
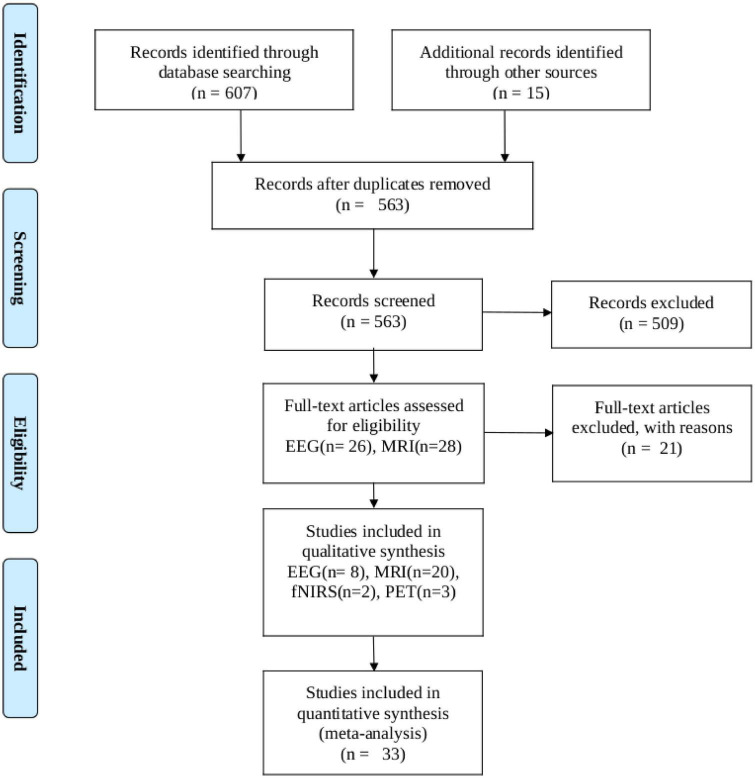
PRISMA flow diagram depicting the study selection process. The search was performed in PubMed up to 12 March 2023.

We conducted a PubMed search up to March 2023, entering ‘thalamotomy’ ablation surgery’ in combination (AND) with the following search terms and their corresponding abbreviations: functional, magnetic resonance (fMRI), electroencephalogram (MRI, and EEG), positron emission tomography (PET), single-photon emission computed tomography (SPECT), Magnetoencephalography (MEG), Functional near-infrared spectroscopy (fNIRS), Electrocorticography (ECoG) and was restricted to articles published in English. We followed Preferred Reporting Items for Systematic Reviews and Meta-analysis (PRISMA) guidelines for meta-analysis (Moher et al., 2009). The PRISMA of the included search results is presented in Figure 1. All data included in this study were related to human diseases that used thalamotomy surgery as a clinical approach. Both unilateral and bilateral thalamotomy were considered. The following inquiries were our focus: We only included articles that met the following criteria: (1) they were written in English, (2) they measured functional connectivity or activity, (3) they involved human subjects, and (4) they provided quantitative or semiquantitative functional activity and connectivity data analyses before and after thalamotomy surgery. We excluded case reports, method articles, case series, and research papers that primarily focused on therapeutic interventions, such as thalamotomy, MRI-guided focused ultrasound, or deep brain stimulation (DBS) and pre-surgical planning.

## Results

Our search turned up 607 results. Zotero (V6) reference manager imported the obtained references, and duplicates were removed. The validity of each title and abstract was verified separately by the authors. By searching through the references of the approved papers, one more paper was found that matched the criteria. In total, 33 publications met our inclusion criteria, with 20 of them being studies that used fMRI, 8 using EEG, 2 using fNIRS, and 3 using PET investigations.

A flowchart of the selection process is presented in Figure 1. The main result and analysis of the included EEG, fMRI, fNIRS, and PET studies are all summarized in Table 1, and the demographic data are summarized in Table 2. There more evidences for fMRI, EEG as non-invasive recordings, which are methods that have been used more than the others in the clinic.

**TABLE 1:** This table summarized functional connectivity studies during thalamotomy surgery.

References	Method	Main results
**fMRI**		
Jang et al., 2016	GTM: FC and pFC	Regulate motor network functional connectivity.
Park et al., 2017	SpDCM	Effective connectivity change in Motor network (VIM).
Hesselmann et al., 2006	pre- and postoperative contrast	Increase of contralateral VIM activity.
Stanziano et al., 2022	ROI to ROI Connectome analysis, and regression model	Increase in rs-FC between bilateral M1 cortices, bilateral M1, and crossed PMC. Decrease in FC between the ACC and bilateral SMA.
Tuleasca et al., 2018f	Group-level ICA	Head tremor arrest following thalamotomy is associated with IC strength between the left SMA and the bilateral thalamus and limbic system.
Tuleasca et al., 2021	ICA, seed to voxel connectivity.	Motor network, cerebellar lobule six are interconnected with visual extrastriate cortex clusters.
Miyagishima et al., 2007	Voxel by voxel analysis	Decrease activity in the Fundus of CS with and without wrist movement.
Tuleasca et al., 2018a	Group-level ICA	Motor, visual and attention network activity are correlated with TSTH score during Vim ablation (left lesion).
Tuleasca et al., 2018d	Temporal correlations to measure FC	Increased FC of sensory-motor and salience networks, left visual association cortex, left superior parietal regions, and decreased FC with the cerebellum.
Tuleasca et al., 2020	Measure dynamic FC using CAP analysis	Decreased in “cerebello-visual-motor,” and increased in “thalamo-visuo-motor,” “basal ganglia and extrastriate” networks comparing HC.
Tuleasca et al., 2018c	Seed to voxel FC using correlations analysis	Decrease in FC between left VLV and right visual areas.
Tuleasca et al., 2018b	Group-level ICA, and correlation analysis	M1 interconnected with the IO nucleus, bilateral thalamus interconnected with motor cerebellum lobule V, anterior DMN interconnected with area 10 (TSTH).
Tuleasca et al., 2018e	Group-level ICA, and correlation analysis	Predict volumes of signature (positive correlation) by inter-connectivity of the anterior-DMN, bilateral thalamus with the motor cerebellum lobule V.
Wen et al., 2016	Voxel by voxel time series similarity, using ReHo change by KCC, correlation analysis.	Decrease ReHo in the cerebellum vermis, MFG, STG, right Hipp, right Thalamus, left cerebellum and increase ReHo in SFG, precentral Gyrus, MTG, ACC, left Thalamus (right lesion).
Lu et al., 2022	fALFF , Seed-based FC.	Increase FC between the right SMG, right SPG, and left precentral gyrus. increased fALFF and RH in the PoCG.
Xiong et al., 2022a	BOLD signal, fALFF.	Decrease in the left occipital visual area activity (left vim).
Xiong et al., 2022b	OrT/CVA	The network associated with ET displayed heightened SMN activity and reduced activity in the posterior cingulate cortex.
Pae et al., 2023	Graph independent component analysis	reduction in the FC of the cerebellum, basal ganglia, and thalamus. Increase in FC premotor cortex and SMA.
Middlebrooks et al., 2023	Whole-brain R-maps	The highest correlation with tremor improvement were found in a nodes within the cerebello-thalamo-cortical pathway, primary visual cortex, and extrastriate visual cortex.
Kato et al., 2022	Dual regression analysis, (FC)	The increase in FC of the SMN and VSN regions.
**EEG**		
Sitnikov et al., 2016	Microelectrode analysis	Increase in alpha rhythm and increased amplitude of the complexes in the parietal, occipital leads.
Jeanmonod et al., 2012	ICA, spectral analysis	Reduction of spectral overactivity, and spectral power in delta and theta bands (frontal, centrotemporal, and parietal regions) and alpha and beta band (centroparietal regions).
Michels et al., 2011	ICA	HPR: decrease in spectral power decrease in spectral overactivity LPR: remaining enhanced spectral power remaining spectral overactivity in IC, lPFC, mPFC, MCC, ACC, PCC, and OFC.
Roth et al., 2000	Power spectral analysis, Coherence analysis	Increase in theta power (both PD and pain groups) Increase in delta power (both Parkinson’s disease) Alpha peak shifted to lower frequencies. the effect diminished three months later.
Sarnthein et al., 2006	Discriminant analysis, Bicoherence analysis	decrease in theta power (measured 3 and 12 months after surgery) reduction of inter-frequency coupling.
Magara et al., 2022	Power spectral analysis,	Increased cortical activity in the delta, theta, alpha, and beta bands. Increased cortical activity across both brain hemispheres and fronto-temporo-insular, frontopolar, OF, and dlPFC regions, cingulate regions.
Stern et al., 2006	power spectral analysis	Decreased overactivation in rACC and midCC.
Choi, 1978	EISA	EEG-driving response diminished in the first postoperative week for low-frequency stimuli but increased after a while.
**fNIRS**		
Kong et al., 2020	paired *t*-tests correlation analysis	Increase in the left M1 and somatosensory cortices (channel 6) and the right dlPFC (channel 45) during the writing task.
Gurgone et al., 2021	GLM analysis	Increase in HbO and HbR concentrations in S1.
**PET**		
Verger et al., 2018	IRCA	A decrease in left thalamic metabolism, right cerebellum, left temporal gyri, and bilateral frontal gyri. stronger connectivity between the left thalamus and right temporo-occipital area prior to treatment.
Henselmans et al., 2000	FDG uptake	Increased metabolic activity in the contralateral pulvinar, Hipp, and ventral mesencephalon, decreased metabolic activity ventrolateral nucleus of the thalamus in the medial region, SMA, and ipsilateral inferior parietal cortex and inferior frontal gyrus show.
Boecker et al., 1997	rCBF changes	A reduction in rCBF, decrease in resting rCBF.

“References” determine the study, “Method” determine the method of functional connectivity analysis, and” Main Results” determines the main finding of the study. Each method of functional connectivity method “fMRI, EEG, fNIRS, and PET” is divided with gray rows. ReHo, regional homogeneity; KCC, Kendall’s coefficient concordance; BOLD, blood oxygen level-dependent; CAP, coactivation pattern; EISA, EEG Interval Spectrum Analysis; IRCA, Graph-theory measures GTM, interregional correlation analysis; spDCM, Spectral dynamic causal modeling; FC, functional connectivity; pFC, partial-correlation functional connectivity; rCBF, regional cerebral blood flow; fALFF, fractional amplitude of low-frequency fluctuation; HbO, oxygenated; HbR, deoxygenated; PoCG, postcentral gyrus; FPG, fludeoxyglucose; TSTH, tremor score on the treated hand; ROIs, regions of interests; IC, interconnectivity; CS, central sulcus; ADL, activities of daily living; MFG, middle frontal gyrus; SFG, frontal gyrus; STG, superior temporal gyri; MTG, middle temporal gyrus; ACC, anterior cingulum; OrT, ordinal trends; CVA, canonical variates analysis; SMN, sensorimotor network; VSN, visuospatial network; HPR, high pain relief; LPR, low pain relief; IC, insular cortex; PCC, posterior cingulate cortex; PFC, prefrontal cortex; mPFC, medial PFC; lPFC, lateral PFC; ACC, anterior cingulate cortex; MCC, midcingulate cortex; OFC, orbitofrontal cortex; rACC, rostral anterior cingulate; MidCC, midcingular cortex; PMC, primary somatosensory cortices; IO, inferior olive; Hipp, hippocampus; SMG, supramarginal gyrus; SPG, superior parietal gyrus; RH, regional homogeneity.

**TABLE 2:** This table summarized the demographic and clinical details of the subjects.

References	Subjects	Lesion area (l/r/both side)	Age (mean ± SD)	Clinical evaluation
**fMRI**				
Jang et al., 2016	ET:8	VIM-MRgFUS (left)	65	CRST
Hesselmann et al., 2006	ET:1	VIM (right)	48	–
Stanziano et al., 2022	TD-PD:15	VIM-MRgFUS (both)	64 ± 7	MDS-UPDRS (part-III)
Tuleasca et al., 2021	ET,PD:100 +	VIM-GK (unilateral)	18∼80	ADL, FTM, TETRAS, QUEST
Miyagishima et al., 2007	ET:6	VIM (left/right)	53.5 ± 16.7	FTM
Tuleasca et al., 2020	ET:15 HC:12	VIM-SRS-T (left)	ET:70 HC:69.4	ADL, TSTH, QUEST
Xiong et al., 2022a	PD:9 HC:9	VIM-MRgFUS (left/right)	PD:64.7 ± 6.1 HC:60.1 ± 6.7	CRST
Tuleasca et al., 2018a,b,c,d,e,f	ET:17 HC:12	VIM-SRS-T (left)	70.1 ± 9.8	ADL, TSTH, QUEST, Head-tremor
Wen et al., 2016	PD:26 HC:31	VIM (left/right)	HC:59.6 ± 7.65 rPD:60.8 ± 7.02lPD:61.4 ± 7.77	mUPDRS, MMSE
Lu et al., 2022	ET:30	VIM-MRgFUS	61.97 ± 10.77	CRST
Xiong et al., 2022b	ET:24	VIM-MRgFUS (left)	61.17 ± 11.49	CRST
Pae et al., 2023	ET:85	MRgFUS	–	–
Middlebrooks et al., 2023	PD:27	Stereotactic (both)	–	TRS
Kato et al., 2022	ET:15 HC:15	MRgFUS (left)	–	CRST
**EEG**				
Sitnikov et al., 2016	Epilepsy:13	ANT (both)	22∼48	Seizure, Engel, ILAE
Jeanmonod et al., 2012	NP:12	CLT-tcMRgFUS (left/right)	45∼75	VAS, Pain
Michels et al., 2011	NP: 23 HC:15	CLT (left/right/bilateral)	38∼71	VAS, QOL
Roth et al., 2000	NP:3 PD:3	MT-PTT (left/bilateral)	46∼65	Sleep, TST
Sarnthein et al., 2006	NP:7	CLT (left/right)	38∼71	VAS, Pain
Magara et al., 2022	CCH:1	CLT-MRgFUS	66	–
Stern et al., 2006	NP:16	CLT (left/right/both)	63 ± 10	VAS
Choi, 1978	PD:67	- (unilateral)	40∼77	–
**fNIRS**				
Kong et al., 2020	ET:7	VIM-tcMRgFUS (unilateral)	63 ± 4.24	CRST, Finger-to-noise, writing task
Gurgone et al., 2021	PD:5 HC:3	MRgFUS (right)	71.8 ± 7.05	Finger tapping
**PET**				
Verger et al., 2018	PD:42 HC:31	VIM-GK (left)	71.6 ± 5.9	MDRS, FTM
Henselmans et al., 2000	PD:4, ET:1 Hemichorea:1	VIM (left, right)	57 ± 15	–
Boecker et al., 1997	PD:2, HC:6	VIM (left)	66, 50	H&Y, UPDRS

“References” determine the reference study, “Year” determine the year when the study got published,” Subjects” determines the number of subjects and their state, “Lesion area” determine the area of ablation, “Age” is the mean and standard deviation on the age of subjects, “Clinical evaluation” determine any clinical evaluation were done in the study. Each method of functional connectivity method “fMRI, EEG, fNIRS, PET” is divided with gray rows. ET, essential tremor; VIM, ventral intermediate nucleus; MRgFUS, magnetic resonance guided focused ultrasound; GK, gamma knife; Stereotactic, open surgery with radiofrequency; CRST, linical Rating Scale for Tremor; MDS-UPDRS, Movement Disorder Society Unified Parkinson’s Disease Rating Scale; TD-PD, Tremor-dominant PD; FTM, Fahn-Tolosa-Marin Clinical Rating Scale for Tremor; TETRAS, Tremor Research Group Essential Tremor Rating Assessment; QUEST, quality of life questionnaire; ILAE, International League Against Epilepsy; Engel, Engel scale; MMSE, Mini-Mental State Examination; QOL, quality of life; TST, total sleep time; VAS, visual analog scale; mUPDRS, unified Parkinson’s disease rating scale motor score connectivity; H&Y, Hoehn and Yahr scale; CCH, chronic cluster headache; CLT, central lateral thalamotomy; PTT, pallidothalamic tract.

### Functional magnetic resonance imaging

The fMRI is a technique used in ablation surgery research to determine the target location of ablation before surgery. Thalamotomy surgeries can affect both structural (Benito-Leon et al., 2018) and functional connectivity within brain network. While several studies have investigated structural alterations within the brain (sub) networks before and after thalamotomy surgery [for review see Bolton et al. (2022)], only a few studies investigated the functionality of the brain network before and after surgery. In this regard, this review article summarizes studies that focused on functional connectivity and activity of the brain intervened by thalamotomy (Table 1). The changes have been followed several times after the surgery to examine the reversibility. Mostly, three brain networks were mostly affected by thalamotomy surgery such as motor, visuomotor, default-mode networks.

The motor network, which is highly involved in tremor symptoms, is primarily affected by thalamotomy surgeries (Xiong et al., 2022a). There is a complex neural mechanisms underlying tremors which involve multiple brain regions and their interconnectivity. Thalamotomy has temporarily reconfigured the whole brain network and resulted in a reduction in the average connection among the motor network (Jeanmonod et al., 2012; Stanziano et al., 2022). Using cross-correlation and partial-correlation methods, Jang et al. (2016) observed an immediate increase inter-hemispheric similarity which reverted after 3 months, and no significant change in the direct connection between the thalamus and the motor cortex area. Moreover, thalamotomy caused selective and consistent changes in effective connectivity from the ventrolateral nuclei and the supplementary motor area (SMA) to the contralateral dentate nucleus (Park et al., 2017). Additionally, a reduction of the activity in the sensorimotor cortex and SMA is observed after thalamotomy (Miyagishima et al., 2007). The interconnectivity strength between the bilateral thalamus and limbic system with SMA predicted head tremors. So, the SMA proved to have a role in the motor network, indicating its effect on modulating head tremors through the abnormal connectivity in the thalamo limbic system (Tuleasca et al., 2018f). Moreover, some resting-state fMRI and task-based fMRI (T-fMRI) studies show no significant alteration in sensorimotor cortex (Park et al., 2017; Xiong et al., 2022a), and just the cingulate motor area is considerably activated (Hesselmann et al., 2006).

It seems that the visuomotor network can be affected by thalamotomy surgery too, according to studies by Kato et al. (2022), Tuleasca et al. (2018c), and Xiong et al., 2022a. They observed changes in functional connectivity within salience and bilateral motor networks as well as within areas involved in hand movement planning or language production (Tuleasca et al., 2018d). Moreover, these studies found interconnection between the ventral intermediate nucleus (VIM) and both the primary motor cortex and the contralateral cerebellum inconsistent with the adjustment role of VIM thalamotomy in the extra-pyramidal circuit (Hyam et al., 2012; Klein et al., 2012). Finally, both structural and functional neuroimaging suggested that the motor network and cerebellar lobule six are interconnected with visual extrastriate cortex clusters (Tuleasca et al., 2021). This suggests that visomotoor integration is complex and may involve multiple brain regions, including those that are targeted by thalamotomy surgery. Tuleasca et al. (2020) applied coactivation pattern (CAP) analysis to characterize three CAP groups: including cerebello-visuo-motor; thalamo-visuo-motor and, basal-ganglia and extrastriate cortex. They observed a decrease in cerebello- visuo-motor occurrence in pretherapy ET compared to healthy control, while thalamo-visuo-motor, basal ganglia, and extrastriate network increased. They suggested that there is a balance between the cerebellar circuitry and the thalamo-visuo-motor and basal ganglia networks, revealing the visual network’s role in tremor generation and suppression after treatment. In addition, Xiong et al. (2022a) have observed a longitudinal dynamic fractional amplitude of low-frequency fluctuations (fALFF) change in the left occipital cortex (Brodmann area 17) in PD patients after MRgFUS thalamotomy and concluded that the visuomotor networks were involved. Tuleasca et al. (2018c) also attempted to predict clinical responses by examining the relationship between pre-therapeutic left ventro-laterla-ventral (VLV) nucleus functional connectivity with right visual association area, left fusiform gyrus, left posterior cingulate. So, they concluded that visual areas played a significant role in this correlation. Overall, these studies highlight the important role of the visuomotor network in tremor generation and suppression and suggest that thalamotomy surgery can impact its associated brain regions.

It is possible that thalamotomy could cause change in default mode network (DMN) activity. Although further research is necessary to fully understand the potential effect. Wen et al. (2016) and Tuleasca et al. (2018e) have both reported on DMN activity in different ways, providing a starting point for understanding potential changes post-thalamotomy. Tuleasca et al. (2018e) focued on predicting volume size, while Wen et al. (2016) examined regional homogeneity (ReHo) changes. Wen et al.’s (2016) findings indicated that the DMN is convergence point for ReHo alteration in the middle temporal gyrus (MTG), anterior cingulum (ACC), and frontal regions.

These regions are involved in various cognitive and behavioral functions, such as memory retrieval, emotion regulation, and social cognition, which could be affected by thalamotomy (Jang et al., 2016; Xiong et al., 2022a).

To sum up, functional connectivity studies have utilized rs-fMRI or T-fMRI methods to examine brain networks associated with tremor circuitry, focusing on networks such as motor, sensorimotor visuo-motor, salience, and default-mode networks. These studies have found that overall activity and connectivity in the motor-related areas were decreased, suggesting changes inn network activity may be involved in tremor suppression after treatment. Further studies are needed to better understand the impact of thalamotomy on the various brain networks and associated cognitive and behavioral functions.

### Electroencephalograms

The electroencephalogram (EEG) is a tool used to study functional connectivity and activity in the brain by measuring the power spectra and phase-locking activity at varying frequency bands in different brain regions. Prior to thalamotomy, EEG recordings showed abnormalities related to over-activity in high theta and low beta frequency bands (Stern et al., 2006). After thalamotomy, changes were observed in the EEG raw signal and power spectrum at different frequency bands, although the effect of on alpha, beta, and theta bands had contradictory results. Some studies reported a decrease in the over-activity in the rostral anterior cingulated cortex (rACC) and midbody of the corpus callosum (midCC) in alpha range (Stern et al., 2006), while others found an increase in alpha power after surgery (Michels et al., 2011). Results regarding beta power were also mixed, with some studies reporting an increase and others finding no change (Michels et al., 2011; Jeanmonod et al., 2012). There were reports of both increased and decreased theta power after thalamotomy surgery (Roth et al., 2000; Michels et al., 2011). It is important to note that these studies were conducted mainly before 2017, and therefore no conclusive evidence has been established to date. In general, activity in all frequency bands tended to decrease after surgery. However, the changes appeared to diminish with time during short post-surgery periods. While low-frequency stimuli initially decreased post-surgery and then increased in the second week (Choi, 1978), changes in power spectra appeared to persist in long post-surgery periods of 3 months to 1 year, lastly total sleep time and sleep efficiency increased after surgery (Roth et al., 2000).

With regard to EEG studies of thalamotomy, the findings are limited by small sample size, methodological differences, and the use of varying outcome measures. Overall, further research is needed to build on the existing knowledge regarding the effect of thalamotomy on EEG activity and functional connectivity, as well as the potential risks and benefits of the procedure in different patient populations.

### Other methods

The more functional connectivity methods commonly used for evaluating thalamotomy surgery include fNIRs and PET. However, their application is limited as fNIRs are only capable of measuring cortical activities and cannot be used to assess deep structures, such as the thalamus and basal ganglia structures. Meanwhile, PET has poor spatial resolution and can only measure brain region activities. Two studies have utilized fNIRs to investigate changes following thalamotomy surgeries, and both studies found an increase in activity in the motor and somatosensory-related areas, as well as functional connectivity with prefrontal areas on the contralateral side (Kong et al., 2020; Gurgone et al., 2021). PET imaging studies have reported a decrease in frontal, parietal, and temporal regions during thalamotomy surgery, and the effect of the surgery on motor-related areas is controversial (Boecker et al., 1997; Henselmans et al., 2000). Furthermore, while one study found a decrease in functional connectivity between thalamus and temporal-occipital regions, however, this decrease diminished 1 week after surgery (Verger et al., 2018).

## Discussion

Thalamotomy has proven to be effective in treating physical symptoms of various diseases such as parkinsonism, hyperkinetic movements, multiple sclerosis, and epilepsy (Spiegel et al., 1947; Ea and Ht, 1950; Elble, 2000; DeLong and Wichmann, 2007; Postuma et al., 2018). However, research examining the influence of thalamotomy on behavioral status is limited. Conducting an analysis on psychological and behavioral data before and after the procedure can help to classify and examine the neurological signs.

The types of surgery, brain recording methods, and brain network analysis influence the functional activity and connectivity results during thalamotomy surgery. Less invasive methods such as MRgFUS (Jang et al., 2016; Stanziano et al., 2022; Xiong et al., 2022a,b; Kato et al., 2022; Pae et al., 2023) and Gamma knife (Tuleasca et al., 2018a,b,c,d,e,f, 2020, 2021; Verger et al., 2018) techniques have emerged, which demonstrate differences in functional connectivity and activity outcomes in comparison with old surgery techniques due to the absence of unwanted tissue ablation in the electrode track.

While EEG was routinely employed to investigate the effects of thalamotomy surgery in the past, fMRI has become more widespread. Combining the both methods in preoperative and postoperative conditions can provide a more thorough examination of the effects of thalamotomy. Using more advanced equipment and greater numbers of electrodes in EEG studies can produce more consistent outcomes, allowing researchers to better understand the indirect impact of thalamotomy surgery on the resulting activity and overlapping functions of brain networks on the human cortex.

Table 1 demonstrates that despite the existence of various voxel-based functional connectivity techniques, such as fALFF (Xiong et al., 2022b), seed-based correlation analysis (SCA) (Jang et al., 2016), and ReHo (Wen et al., 2016), most present EEG and fMRI studies apply a data-driven ICA analysis. While new methods are continuously being developed, the brain is a dynamic and complex system that undergoes significant changes over time, making it a non-stationary network. To better model changes within the network, novel techniques such as Comprehensive Autonomic Predictor (CAP) (Tuleasca et al., 2020), Dynamic Causal Modeling (DCM) (Park et al., 2017), and windowed analysis may be more applicable. For future studies, more hypothesis-based approaches are recommended.

However, in terms of maintaining the brain’s equilibrium and functionality, how do the tremor circuit and other networks collaborate? The tremor circuit is not a subset of a specific network, so it’s possible that other essential contributors are present alongside the precentral gyrus, thalamus, and dentate nucleus. As reported by Miyagishima et al. (2007) the fundus of the central sulcus (Brodmann area 3a) is an additional critical relay in the tremor circuit, receiving proprioceptive inputs from the VIM nucleus. Therefore, a decrease in activation within the sensorimotor cortex and SMA can occur following thalamotomy. Tremors have been found to be associated with functional connection abnormalities in higher-level cortical and visual areas. The network-level functional connectivity changes in cortical, basal ganglia, and cerebellar systems have also been linked to tremors (DeSimone et al., 2019). Thus, among all the studies that have focused on the pathophysiology of essential tremor (ET), the role of the cerebellum’s dentate nuclei has been strongly supported (Jang et al., 2016; Park et al., 2017; Tuleasca et al., 2021; Stanziano et al., 2022). Tuleasca et al. (2020) have proposed a hypothesis that considers the broader impact of the visual system on tremor production. They suggest that if more than two brain networks are activated, it may be difficult to determine which one affects the other. This hypothesis indicates that the visual network could impact the motor network or vice versa. In an attempt to address such questions, Park et al. (2017) and Tuleasca et al. (2020) utilized DCM and CAP, respectively.

Passive movement in the brain is demonstrated by hemodynamic changes through fMRI and PET, while direct synchrony in the brain is depicted by EEG. However, functional association between basal ganglia and motor cortex (Brooks, 1999), as well as the asymmetric effect in postoperative patients by cortical metabolic (Baron et al., 1992), has been confirmed by both fMRI and PET studies. In PET scans comparing thalamotomy and pallidotomy of basal ganglia in pre- and post-surgical cases, the (pre)frontal cortex, lateral prefrontal, and parietal cortex were identified (Henselmans et al., 2000), and these are potential locations for future fMRI studies to track DMN changes. Basal ganglia connect with the SMA, dorsal prefrontal cortex, and frontal association areas. Consequently, insufficient activity in basal ganglia triggers compensatory overactivity of the lateral premotor and parietal cortex, which is primarily responsible for facilitating motor responses to visual and auditory cues. The association of subcortical activity areas that primarily receive input from basal ganglia demonstrates dysfunction of motor and visuomotor network activity in the tremor circuit (Jones, 1985).

Research studies that solely focused on structural changes have revealed alterations in the temporal pole and occipital cortex, indicating the significance of targeting specific visuomotor networks (Wen et al., 2016). Allowing researchers to understand the indirect effect of thalamotomy surgery on the human motor cortex and its impact on the overlapping activity of brain networks. While the majority of studies have focused on network connectivity alterations in imaging results, this hypothesis can be verified by comparing the functional connectivity and activity observed in EEG and fMRI studies in the future. According to Table 2, a common pattern noticed in both EEG and fMRI studies was the decrease-increase pattern reconfiguration in EEG studies (Brooks, 1999; Park et al., 2017) and the pattern evaluated with Clinical Rating Scale for Tremor (CRST) in fMRI studies (Jang et al., 2016; Park et al., 2017; Xiong et al., 2022b).

Studies have demonstrated the impact of thalamotomy on visuomotor network changes (Tuleasca et al., 2018a,c, 2020; Xiong et al., 2022a). According to Table 2, the VAS is a standard clinical evaluation technique utilized in EEG studies (Sarnthein et al., 2006; Stern et al., 2006; Michels et al., 2011; Jeanmonod et al., 2012). This investigation suggests that the effect of thalamotomy on the visual network is based on an earlier pathological belief that thalamotomy alters the visual network. Studies on the thalamus from as early as 1,664 and 1,681 by Thomas Willis described the human thalamus as the “chambers of the Optic Nerves.” Luys (1865) reported that thalamic nuclei are correlated with sensory, motor, limbic, and intrinsic functions. Karl Friedrich Burdach, since 1,822, has followed the optic tract and the superior colliculum’s brachium to the lateral geniculate body (Jones, 1985).

Although studies have examined various networks, such as the visual network, default mode network (DMN), and motor networks, the role of the mediodorsal thalamic nucleus in olfaction remains an unanswered question, considering that all senses except olfaction pass through the thalamus.

## Author contributions

M-HN, SE, ER, M-RD, A-HV, MSa, AE, MSh, and YB contributed to the study design. M-HN, SE, and ER performed the data collection and analysis. M-HN, SE, ER, M-RD, A-HV, and MSa contributed to manuscript drafting and approved the submitted version. All authors contributed to the article and approved the submitted version.
